# Controlling Coancestry and Thereby Future Inbreeding by Optimum-Contribution Selection Using Alternative Genomic-Relationship Matrices

**DOI:** 10.3389/fgene.2020.00345

**Published:** 2020-04-22

**Authors:** G. T. Gebregiwergis, Anders C. Sørensen, Mark Henryon, Theo Meuwissen

**Affiliations:** ^1^Department of Animal and Aquaculture Sciences, Norwegian University of Life Sciences, Ås, Norway; ^2^Department of Molecular Biology and Genetics, Aarhus University, Aarhus, Denmark; ^3^Seges, Copenhagen, Denmark; ^4^School of Agriculture and Environment, University of Western Australia, Crawley, WA, Australia

**Keywords:** true inbreeding, genetic gain, genomic optimum contribution selection, genomic relationship matrices, prediction

## Abstract

We tested the consequences of using alternative genomic relationship matrices to predict genomic breeding values (GEBVs) and control of coancestry in optimum contribution selection, where the relationship matrix used to calculate GEBVs was not necessarily the same as that used to control coancestry. A stochastic simulation study was carried out to investigate genetic gain and true genomic inbreeding in breeding schemes that applied genomic optimum contribution selection (GOCS) with different genomic relationship matrices. Three genomic-relationship matrices were used to predict the GEBVs based on three information sources: markers (**G**_M_), QTL (**G**_*Q*_), and markers and QTL (**G**_A_). Strictly, **G**_*Q*_ is not possible to implement in practice since we do not know the quantitative trait loci (QTL) positions, but more and more information is becoming available especially about the largest QTL. Two genomic-relationship matrices were used to control coancestry: **G**_M_ and **G**_A_. Three genetic architectures were simulated: with 7702, 1000, and 500 QTLs together with 54,218 markers. Selection was for a single trait with heritability 0.2. All selection candidates were phenotyped and genotyped before selection. With 7702 QTL, there were no significant differences in rates of genetic gain at the same rate of true inbreeding using different genomic relationship matrices in GOCS. However, as the number of QTLs was reduced to 1000, prediction of GEBVs using a genomic relationship matrix constructed based on **G**_*Q*_ and control of coancestry using **G**_M_ realized 29.7% higher genetic gain than using **G**_M_ for both prediction and control of coancestry. Forty-three percent of this increased rate of genetic gain was due to increased accuracies of GEBVs. These findings indicate that with large numbers of QTL, it is not critical what information, i.e., markers or QTL, is used to construct genomic-relationship matrices. However, it becomes critical with small numbers of QTL. This highlights the importance of using genomic-relationship matrices that focus on QTL regions for GEBV estimation when the number of QTL is small in GOCS. Relationships used to control coancestry are preferably based on marker data.

## Background

Optimum contribution selection (OCS) is a selection method that maximizes genetic gain while controlling inbreeding (Meuwissen, 1997). It does this by optimizing the genetic contribution of selection candidates to the next generation using estimated breeding values and genetic relationships between candidates. A pedigree-based relationship matrix (**A**) was initially used to control inbreeding (Meuwissen, 1997). However, pedigree relationships have limitations. The **A**-matrix measures relationships and inbreeding at neutral, unlinked, and independent loci. But, genomic regions flanking quantitative trait loci (QTL) under selection lose more variation than neutral regions of the genome (Roughsedge et al., 2008). It also does not consider variation due to Mendelian sampling during gamete formation, assuming the same relationship between all full-sibs (Nejati-Javaremi et al., 1997; Avendaño et al., 2005). Dense panels of single nucleotide polymorphism (SNP) markers may be used to trace Mendelian segregation at marker loci (Hayes et al., 2009). Therefore, genomic markers might help to overcome some of the limitations imposed by pedigree.

There are several methods available to calculate genomic-relationships matrices (Nejati-Javaremi et al., 1997; Eding and Meuwissen, 2001; VanRaden, 2008; Yang et al., 2010; VanRaden et al., 2011). They have been used in different settings to realize high accuracies of genomic-prediction of breeding values and increase genetic gain (Jannink, 2010; Gómez-Romano et al., 2016). Moreover, genomic relationships that incorporate QTL information realize higher accuracies of genomic breeding values (GEBVs) than genomic relationships constructed based on markers only (Nejati-Javaremi et al., 1997; Zhang et al., 2010). In case of few QTL (100) and many records, accuracies of GEBVs close to one have been achieved (Fragomeni et al., 2017). Although, adding QTL information in the construction of genomic relationship matrices improved accuracies of prediction, there is no full understanding on the interaction of the use of alternative genomic relationship matrices in genomic optimum contribution selection (GOCS) schemes.

Optimum contribution selection can be extended into GOCS by using genomic information for both prediction of breeding values and estimation of relationship among selection candidates to manage group coancestry and thereby future inbreeding (Sonesson et al., 2012; Woolliams et al., 2015). Although, genomic relationship matrices that are based on dense SNP markers can reflect true relationship between individuals with a high degree of precision (Goddard, 2009), the covariances between additive genetic values of individuals for a specific trait are more accurately estimated using the relationships based on causal loci than SNP markers (Zhang et al., 2010; Habier et al., 2013; Yang et al., 2015). Moreover, there are limitations on our understanding of the way SNP markers are used to control group coancestry and thereby future inbreeding in GOCS (De Beukelaer et al., 2017; Henryon et al., 2019). Hence, our hypothesis is that the use of genomic relationship matrices based on QTL for the prediction of GEBV and marker based genomic relationships for OCS to control coancestry could show synergies regarding the rates of genetic improvement.

In this study, we investigated the use of alternative genomic relationship matrices for the prediction of GEBV and for the management of coancestry, where these relationship matrices are not necessarily identical. We investigated these combinations of relationship matrices by simulating three genetic architectures with 7702, 1000, and 500 QTLs. Alternative genomic relationship matrices were calculated using different genomic information sources such as SNP markers, QTLs, and both SNP markers and QTLs.

## Materials and Methods

We used stochastic simulations of breeding schemes to estimate rates of genetic gain realized by OCS at the same rate of true inbreeding with three matrices for prediction and two matrices to control of coancestry using three genetic architectures. The three prediction matrices were constructed using genetic markers (**G**_M_), QTL (**G**_*Q*_), and markers and QTL (**G**_A_). The two matrices to control coancestry were **G**_M_ and **G**_A_. **G**_*Q*_ was not used to control coancestry because allele frequency changes at the QTL were assumed desirable (increasing positive allele frequencies) in order to realize genetic gains. The six alternative genomic relationship matrix combinations for prediction and control of coancestry were A_A (both prediction and control of coancestry use **G**_A_), M_A (prediction of GEBV using **G**_M_ and control of coancestry using **G**_A_), Q_A (prediction of GEBV using **G**_*Q*_ and control of coancestry using **G**_A_), A_M (prediction of GEBV using **G**_A_ and control of coancestry using **G**_M_), M_M (both prediction and control of coancestry using **G**_M_), and Q_M (prediction of GEBV using **G**_*Q*_ and control of coancestry using **G**_M_). We investigated three genetic architectures with very many QTL (7702), a large number of QTL (1000), and a moderate number of QTL (500). For comparison, Wood et al. (2014) found that ∼2000, ∼3700, and ∼9500 SNPs explained ∼21, ∼24, and ∼29% of phenotypic variance of human height. QTL were randomly positioned on the genome of 18 chromosomes of equal length (167 cM), which resembles the pig genome.

### Simulation of Genome and Population

#### Founder Populations

**FIGURE 1:**
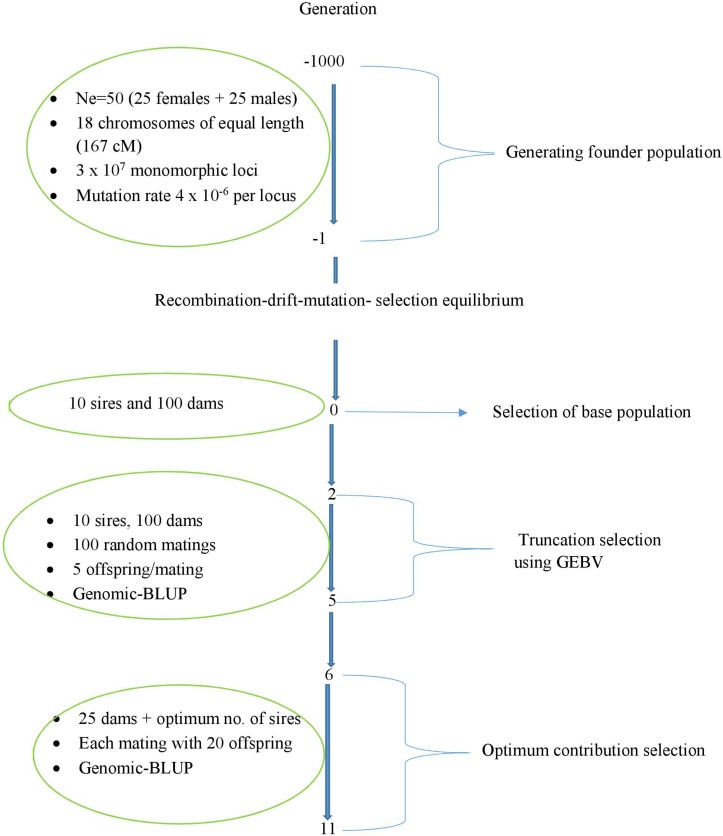
Schematic representation of the simulated breeding scheme.

A schematic representation of the simulated breeding scheme is presented in Figure 1. Using 25 males and 25 females, a founder population was initiated and simulated for 1000 discrete generations. And, the effective-population size (Ne) of 50 was kept constant in each generation. The founder population genomes consisted of 3006 cM contained 30,000,000 equidistant monomorphic loci (both markers and QTLs; 1 × 10^4^ loci per cM). The ratio of QTL loci to marker loci was 1:7. As a result 1/8 of the monomorphic loci (3.75 × 10^6^) were QTL loci and the remaining loci were SNP markers. The mutation rate was assumed to be 4 × 10^–6^ per locus in order to generate bi-allelic polymorphism at mutated loci. The number of recombination sites for the *i*th animal was sampled from a Poisson distribution as *n*_Q*i*_ = Pois (λ), where λ = 30.06 is the length of the genome in Morgans. The *n*_Q*i*_ recombination sites were placed randomly on the genome.

Recombination-drift-mutation-selection equilibrium of the founder population was reached after 1000 generations. Moreover, linkage disequilibrium between the QTL and markers alleles was established during the simulation of the founder population with a Fisher–Wright inheritance model (Fisher 1930, Wright 1931).

In each generation, male and female parents of the next generation were randomly sampled with replacement from the 25 males and 25 females of the current generation. The additive-genetic effect of the original allele at each QTL locus was set to zero. The additive-genetic effect of the mutant allele at each QTL was sampled from an exponential distribution and it was assumed to be positive with a probability of 0.1. Selection was introduced by culling and resampling approximately 5% of animals with the lowest true breeding value (TBV) in each generation.

The TBV of an individual in the founder population was calculated as:

${\text{TBV}}_{i}=\sum_{j=1}^{n}\left({\text{x}}_{ij}{\text{g}}_{j}\right)$, where *n* is the number of QTL across the genome in the *i*-th founder animal, x_*ij*_ is the number of copies of the mutant allele that animal *i* inherited at the *j*-th QTL (x_*ij*_ = 0, 1, 2), and g_*j*_ is the additive effect of the mutant allele at the *j*-th QTL.

The average decay of linkage disequilibrium with distance between the segregating marker loci in generation 1000 of the founder population was similar to the average decay seen in the three commercial breeds of Danish pigs (Wang et al., 2013).

The founder population had 61,920 (7702 QTL and 54,218 marker) segregating loci at generation 0. All 54,218 segregating marker loci were used in our breeding schemes. The number of segregating QTL used in the breeding schemes were all segregating QTLs (7702 QTL), 1000 or 500 QTLs. The number of segregating QTLs across the genome were reduced to a desired number, i.e., 1000 QTLs or 500 QTLs by random sampling from the 7702 QTLs.

The additive-genetic effects of the segregating mutant QTL alleles (7702, 1000, or 500) were standardized in order to get a total additive–genetic variance of 1 for the trait under selection in the founder population at generation 0.

We sampled a population of 100 chromosomes of chromosome number 1, representing the chromosome 1 chromosomes of 50 animals (two chromosomes per animal). The same sampling was performed for chromosome 2, 3, …, 18. These 50 animals formed the founder generation 0.

#### Base Populations

In each replicate of the simulation, a unique base population with a size of 110 animals (10 males and 100 females) was sampled from the pools of chromosomes of the founder population to initiate the breeding schemes. The genotype of each base animal was sampled from the 18 pools of chromosomes in the founder population. For chromosome *j* (*j* = 1 … 18), two chromosomes were randomly sampled without replacement from the *j*th pool of 100 chromosomes. The sampled chromosomes were replaced before the next base animal was sampled. Base animals were assumed unrelated and non-inbred based on pedigree and identical by descent (IBD) alleles. They were genotyped, but not phenotyped.

### Identical by Descent Markers

A total of 12,024 IBD loci were used to measure the true rate of inbreeding (ΔF_true_) and were placed evenly across the genomes in the base population at 4 IBD loci per cM. Base animals were assigned unique alleles at each IBD locus (i.e., 2*n* distinct alleles at each IBD locus among the *n* animals in the base population), such that identical alleles in any later generation indicates that the loci are IBD with a single unique base population allele. The IBD loci were not involved in any way in the selection.

### Simulation of Phenotypic Values

Phenotypic value of animal *i*, P_*i*_, were simulated as: *P*_*i*_ = TBV_*i*_ + *e*_*i*_, where *e*_*i*_ is an error term for individual *i* sampled from $e_{i}∼N\left(0,\sigma_{e}^{2}=4\right)$ resulting in a trait heritability of 0.2; and TBV_*i*_ is the TBV of animals which was obtained as described above.

### Genomic Estimated Breeding Values

The G-BLUP model (Meuwissen et al., 2001) was used to predict GEBVs:

(1) y=1μ+Zg+e,

where *y* is a vector of phenotypes, μ is the overall mean, 1 is a vector of ones, *Z* is a design matrix allocating records to breeding values, *g* is a vector of breeding values for all animals with $\text{Var}\left(g\right)=G\sigma_{g}^{2}$, G is the genomic relationship matrix, and $\sigma_{g}^{2}$ is the additive genetic variance. The term *e* is a vector of normal independent and identical distributed residuals with variance $\sigma {}_{g}^{2}$.

### Genomic-Relationship Matrices

Genomic relationship matrices (*G*) were computed using VanRaden method 2:

$$G=\frac{WD^{-1}W\prime }{L},$$

where *W* is a matrix of centered marker genotypes by subtracting the mean of the marker or QTL genotypes; *L* is the number of loci; *D* is a diagonal matrix with entries 2*p*_*i*_ (1−*p*_*i*_); and *p*_*i*_ is frequency of the minor allele at locus *i* in the base population. All animals in the base population were used to calculate *p*_*i*_ to center and scale genotypes at locus *i*. After scaling, each locus obtained equal weight. The prediction and control of coancestry matrices, **G**_M_, **G**_*Q*_, and **G**_A_, were constructed using marker, QTL, and marker and QTL genotypes, respectively.

### Truncation Selection

The base population animals were randomly mated to produce 500 offspring with equal sex ratio in generation 1. A truncation selection breeding program was conducted in generation 2–5 in order to mimic a population that had undergone selection. Ten sires and 100 dams were truncation selected based on genomic-estimated breeding values. Each selected sire was randomly mated with 10 dams and each mating produced five offspring. As the result of these matings, 500 offspring with an equal sex ratio were obtained.

### Optimum Contribution Selection

Evolutionary algorithms (EVA) was used to optimize individual genetic contributions by maximizing the function *U*_t_ with respect to **c**:

(2) $$U_{\text{t}}\left(c\right)=c\prime \hat{g}-\omega c\prime \mathrm{Gc},$$

where **c** is a *n* vector of genetic contributions of the current generation to the next which is proportional to the number of offspring each animal obtains; $\hat{g}$ is a *n* vector of genomic estimated breeding values, ω is a penalty applied on the average relationship of the selected parents for the next generation, and **G** is a *n* × *n* genomic relationship matrix among all animals in the population calculated as **G**_M_ or **G**_A_. In the above function, $c\prime \hat{g}$ and *c*′*Gc* represent the average genetic value and average relationship of the new generation. For a detailed description of the EVA method see Henryon et al. (2015).

Optimum contribution selection was carried out in generations 6–11. A total of 25 matings were allocated to 500 selection candidates (approximately 250 males and 250 females) by OCS in each generation. Each male was allocated 0, 1, 2, …, or 25 matings in correspondence to their optimum contributions, **c**. Each of 25 selected female was allocated a single mating. The 25 sire and dam were mated randomly. Each mating produced 20 offspring, resulting in 25 full-sib families and 500 offspring. Offspring were assigned as males / females with a probability of 0.5.

### Data Analyses

We plotted the rate of genetic gain against the rate of true inbreeding at different penalties (ω) for the schemes with 7702 and 500 QTL. The ω-values used were −50, −25, −10, and −5. For the scheme with 1000 QTL, we presented rates of genetic gain at 1 and 0.5% rates of true inbreeding. The 1 and 0.5% rates of true inbreeding were realized by calibrating the penalty, ω, in Eq. 2. We also compared the accuracies of males and females selection candidates for 1000 QTL at 1% rate of true inbreeding. Rates of genetic gain were calculated as the slope of the linear regression of *G*_t_ on *t* where *G*_t_ is the average true genetic value of animals born in generation *t* (*t* = 6 … 11). Rates of true inbreeding (using the IBD markers) and rates of inbreeding based on pedigree were calculated as 1-exp(β), where β is a linear regression of ln(1−F_t_) on *t* and F*_t_* is the average coefficient of true inbreeding or pedigree inbreeding for animals born at generation *t* (*t* = 6 … 11) (Sonesson et al., 2005). F_t_ for true inbreeding was calculated using the *d* = 12,024 IBD markers as $F_{\text{t}}=\frac{1}{n_{\text{t}}d}\sum_{i=1}^{n_{\text{t}}}\sum_{j=1}^{d}\delta_{ij}$, where *n*_t_ is the number of animals born in generation *t* and δ_*ij*_ is the IBD status at IBD-marker locus *j* (*j* = 1 … *d*) for animal *i* (*i* = 1 … *n*_t_). δ_*ij*_ was equal to 1 if the alleles at IBD locus *j* for animal *i* were identical for a unique base-allele, and 0 otherwise.

### Software

The simulations were run using the program ADAM (Pedersen et al., 2009). BLUP-breeding values were estimated using DMU6 (Madsen et al., 2006). OCS was carried out by EVA (Berg et al., 2006).

## Results

### 500 QTL

**FIGURE 2:**
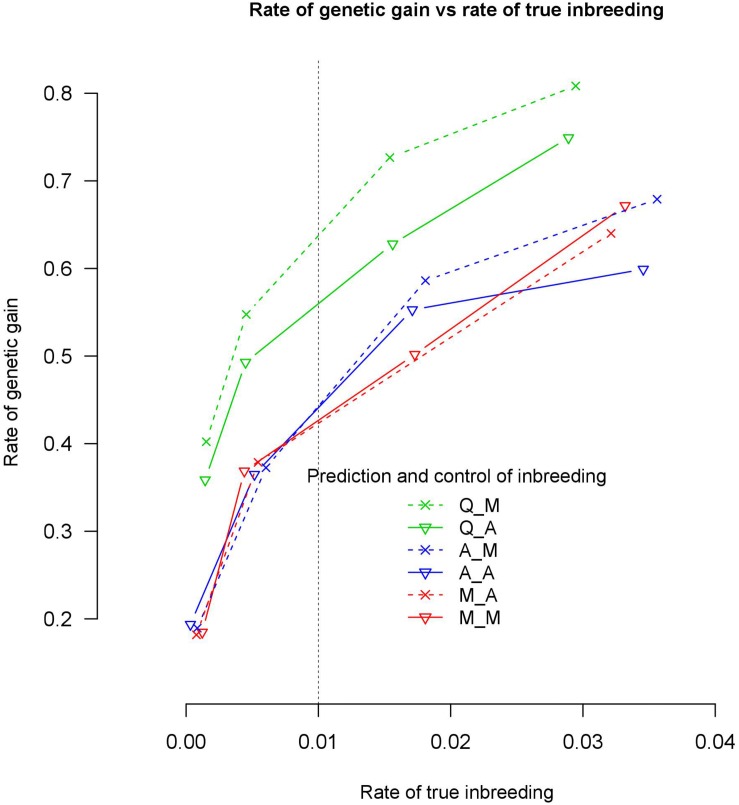
Rate of genetic gain and true inbreeding (IBD) using 500 QTLs in genomic optimum contribution selection.

Quantitative trait loci based genomic relationship matrices for prediction of breeding values with marker based genomic relationship matrices to control coancestry, Q_M, realized more rate of genetic gain at a true inbreeding rate of 0.01 than the 5 alternative methods tested in this study (see Figure 2).

### 1000 QTL

Quantitative trait loci based genomic relationship matrices for prediction of breeding values with marker or all loci based genomic relationship matrices to control coancestry, Q_M and Q_A, realized more rate of genetic gain at a 1% rate of true inbreeding than 4 alternative methods of G-matrices. Q_M and Q_A realized between 21.5 and 29.7% more genetic gain than A_M, A_A, M_M, and M_A at 1% rate of true inbreeding (Table 1). At 0.5 % rate of true inbreeding, it realized between 29.9 and 53% more genetic gain than A_M, A_A, M_M, and M_A. Q_M realized almost the same rate of genetic gain as Q_A with both 1 and 0.5% rate of true inbreeding. Use of genomic relationship matrices computed based on QTLs (**G**_*Q*_) to predict GEBVs gave higher accuracy of prediction than **G**_M_ or **G**_A_ at 1% rate of true inbreeding (Table 2). The accuracy of prediction of male selections using Q_M was 12.7% higher than M_M at 1% rate of true inbreeding (Table 2). This implies that the improvement in accuracy (12.7%) of the GEBVs explained only part of the increased in genetic gain (29.9%), i.e., 43% (12.7/29.9) of genetic gain was due to improvement in accuracy of GEBVs.

**TABLE 1:** Rate of genetic gain and rate of pedigree based inbreeding in genomic optimum contribution selection using different genomic information to predict GEBV and control of coancestry.

G-matrices	ΔIBD = 0.01		ΔIBD = 0.005	
	ΔG(SE)	ΔF (SE)	ΔG (SE)	ΔF (SE)
Q_M	0.677 (0.005)	0.012 (0.0002)	0.586 (0.007)	0.0086 (0.0003)
Q_A	0.672 (0.005)	0.012 (0.0002)	0.586 (0.006)	0.0085 (0.0003)
A_M	0.537 (0.005)	0.012 (0.0002)	0.433 (0.006)	0.0092 (0.0002)
A_A	0.544 (0.004)	0.012 (0.0002)	0.401 (0.005)	0.0093 (0.0003)
M_M	0.522 (0.005)	0.012 (0.0003)	0.383 (0.006)	0.0085 (0.0003)
M_A	0.557 (0.005)	0.012 (0.0002)	0.451 (0.007)	0.0086 (0.0003)

*Rates of true inbreeding were 1 and 0.5% and there were 1000 QTL. ΔG, rate of genetic gain; ΔIBD, rate of true inbreeding; ΔF, rate of inbreeding based on pedigree; and SE, standard errors based on 100 replicates.*

**TABLE 2:** Accuracy of GEBVs for alternative genomic relationship matrices used to predict GEBVs and control of coancestry in genomic optimum contribution selection in generation 11 and at a rate of true inbreeding of 0.01.

G-matrices	Male accuracy (SE)	Female accuracy (SE)
Q_M	0.728 (0.005)	0.738 (0.005)
Q_A	0.721 (0.005)	0.720 (0.005)
A_M	0.661 (0.005)	0.661 (0.005)
A_A	0.656 (0.005)	0.660 (0.005)
M_M	0.646 (0.006)	0.646 (0.006)
M_A	0.659 (0.006)	0.659 (0.005)

*The number of QTLs = 1000.*

### 7702 QTL

**FIGURE 3:**
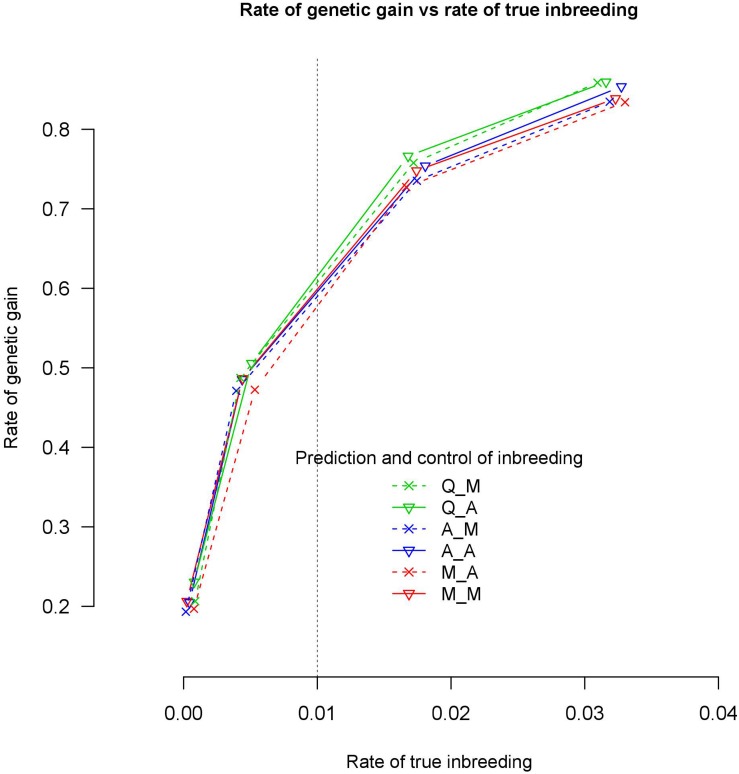
Rate of genetic gain and true inbreeding (IBD) using 7702 QTLs.

With 7702 QTL, the six genomic relationships matrix combinations for prediction and control of coancestry realized almost the same rate of genetic gain at the same rate of true inbreeding (Figure 3). However, with an increase of the rate of true inbreeding >1%, the differences between the rate of genetic gain obtained by the different genomic relationship matrices became visible and the rate of genetic gain of Q_M and Q_A became slightly higher than the other four genomic relationships matrix combinations. However, all these differences in rate of genetic gain at the same rate of true inbreeding were not significant at 95% confidence intervals.

## Discussion

Our findings partly supported our hypothesis that prediction with QTL and control of coancestry with markers, Q_M, realizes more genetic gain at the same rates of true inbreeding than prediction and control of coancestry with markers, M_M. We found that prediction with QTL and control of coancestry with markers realized more genetic gain when 500 and 1000 QTL controlled the trait under selection. However, when the trait was controlled by 7702 QTL, prediction and control of coancestry with markers realized just as much genetic gain as prediction with QTL and control of coancestry with markers. These findings are important because they highlight that when traits under selection are controlled by small numbers of QTL, we need to select directly for the QTL to maximize genetic gain at pre-defined rates of true inbreeding. This implies that we need to know the exact number of QTLs controlling the trait. On the other hand, we do not need to select directly for the QTL to maximize genetic gain when traits are controlled by large numbers of QTL. In this scenario, prediction using markers that are in linkage disequilibrium with the QTL (and control of coancestry using markers in LD with IBD alleles) is sufficient. Therefore, the method used in prediction and control of coancestry when using OCS depends on the number of QTL controlling the trait under selection.

Prediction with QTL and control of coancestry with markers only realized more genetic gain when 500 and 1000 QTL controlled the trait under selection for two reasons. First, prediction with QTL was more accurate than prediction with markers when small numbers of QTL controlled the trait. Prediction with QTL generated accurate breeding values because it had perfect knowledge of the true genetic (co)variance among individuals for the trait under selection. Prediction with markers was not as accurate because there was insufficient LD between the markers and QTL. Many of the markers where not located near QTL. With high numbers of QTL, prediction with markers had similar accuracy of prediction as prediction with QTL. There was more LD between the markers and QTL when many QTL were distributed across the genome. The second possible reason was that control of coancestry restricted changes in QTL-allele frequencies less when there were small numbers of QTL controlling the trait under selection. Control of coancestry in GOCS traced and penalized changes in marker-allele frequencies brought about by realized genetic drift and selection (Woolliams et al., 2015). It penalized changes in allele frequencies at all marker loci. Because these marker alleles were in linkage disequilibrium with QTL alleles, it restricted changes in QTL-allele frequencies. Inbreeding control is spread over the whole genome. With few QTL, much of the inbreeding control could be at regions of the genome that do not harbor QTL. With many QTL, inbreeding control is at all regions of the genome that harbor QTL – it penalized changes in allele frequencies at all loci, when we need to allow allele-frequency changes at some QTL loci. Therefore, genomic relationship matrices used to predict GEBV may differ from that used to control coancestry and thereby future inbreeding. In addition, genomic relationship matrices that consider the true genetic architecture of a trait under selection and allow differentiating the inbreeding rates at the QTL from the general rates at the genomic level could realize higher rates of genetic gain at the same rate of true inbreeding.

Prediction with QTL and control of coancestry with markers cannot be implemented directly in practical breeding schemes. However, this scheme does teach us some principles that apply to practical breeding schemes. Genomic relationship matrices based on QTL are not currently available in practice and it is unlikely to be available soon since exact number of QTLs controlling a trait are not known. Although, it is not possible to get a genomic-relationship matrix based on QTL, a trait specific genomic relationship matrix could be available from genome-wide association studies. Previous studies have shown that trait-specific genomic-relationship matrices realize higher prediction accuracy than marker-based genomic-relationship matrices but it realizes lower accuracy of prediction than the QTL-based genomic-relationship matrix (Nejati-Javaremi et al., 1997; Zhang et al., 2010; Fragomeni et al., 2017). Until QTL-based genomic-relationship matrices become available, trait-specific genomic-relationship matrices could be used for prediction. Moreover, the inbreeding can be controlled using a selected panel of markers that has no association with the trait under selection to relax inbreeding control around the QTL regions. Therefore, when a trait under selection is controlled by a small number of QTLs, OCS that incorporates information about the trait for breeding value prediction realizes more genetic gain. Hence, there is an incentive for research work aiming to obtain more biological information about the QTL that underlie the breeding goal traits.

Our findings with 500 and 1000 QTL controlling the trait under selection are supported by several studies that assessed the role of alternative genomic relationship matrices on accuracy of genomic estimated breeding values (Nejati-Javaremi et al., 1997; Zhang et al., 2010; Hickey et al., 2013; Fragomeni et al., 2017). These studies addressed the effects of alternative genomic-relationship matrices on predictions without considering inbreeding control. However, our results are generally supported by their findings as we extended the study toward OCS and assessed both the effects on prediction and on control of coancestry. Fragomeni et al. (2017) showed that, adding causative QTN in an unweighted genomic relationship matrix improved the accuracy of prediction by 0.04. However, using a weighted genomic relationship matrix with weights obtained from genome-wide association studies, they reported an increase of accuracy by 0.1. These findings in general agree with our findings on (500 and 1000) QTL where Q_M realized the highest accuracy of prediction and genetic gain. Moreover, prediction with all loci (both markers and QTL) and inbreeding control with markers, A_M, realized higher accuracy of prediction than M_M. Our results also showed that the difference in rate of genetic gain obtained between Q_M and M_M became smaller as the number of QTL became larger, i.e., 500 and 1000 QTL. Moreover, this difference became insignificant as the number of QTLs further increased to 7702 QTL. Similarly, Nejati-Javaremi et al. (1997) reported higher accuracies and response to selection using genomic relationship matrices constructed based on QTL genotypes when a trait is controlled by a small number of loci (5 vs 25 and 100). Fragomeni et al. (2017) also reported higher accuracy using 100 QTL than 1000 QTL. This implies that the accuracy of prediction of the QTL based genomic relationship matrices increases as the number of QTLs controlling the traits decreases. Therefore, our findings that differences in rate of genetic gain between Q_M and M_M increase as the number of QTL decrease from 1000 to 500 QTLs may hold also for very few QTLs controlling the traits as previous studies reported (Nejati-Javaremi et al., 1997; Fragomeni et al., 2017).

## Conclusion

This study showed that as the number loci involved in the control of the trait of interest are large, genomic relationship matrices based on markers for both prediction and control of coancestry, M_M, perform as good as genomic relationship matrix constructed based on QTLs (Q_M) in GOCS. Whereas, when the trait is controlled by a small number of genes, genomic relationship matrix constructed based on QTLs (Q_M) realize higher rates of genetic gain than genomic relationships constructed based on markers (M_M) at the same rate of true inbreeding in GOCS. This improved rate of genetic gain of Q_M was partly explained by the increased prediction accuracy using **G**_*Q*_, and partly by using a different relationship matrix for prediction as for coancestry control **G**_M_, which creates opportunities for the genomic optimum contribution algorithm to enhance genetic gain.

## Data Availability Statement

The datasets analyzed in this manuscript are not publicly available. Requests to access the datasets should be directed to gebreyohans.tesfaye.gebregiwergis@nmbu.no.

## Author Contributions

GG performed the study and drafted the manuscript. GG, TM, AS, and MH designed the study. MH modified the ADAM program. TM and AS planned and coordinated the whole study. TM and MH contributed to writing the manuscript. All authors read and approved the final manuscript.

## Conflict of Interest

MH is employed at SEGES. The remaining authors declare that the research was conducted in the absence of any commercial or financial relationships that could be construed as a potential conflict of interest.
